# The aging clock and circadian control of metabolism and genome stability

**DOI:** 10.3389/fgene.2014.00455

**Published:** 2015-01-14

**Authors:** Victoria P. Belancio, David E. Blask, Prescott Deininger, Steven M. Hill, S. Michal Jazwinski

**Affiliations:** ^1^Department of Structural and Cellular Biology, Tulane School of Medicine, Tulane UniversityNew Orleans, LA, USA; ^2^Tulane Cancer Center, Tulane Center for Aging, and Tulane Center for Circadian BiologyNew Orleans, LA, USA; ^3^Department of Epidemiology, Tulane UniversityNew Orleans, LA, USA; ^4^Department of Medicine, Tulane UniversityNew Orleans, LA, USA

**Keywords:** aging, light exposure at night, retroelements, LINE-1, metabolism

## Abstract

It is widely accepted that aging is characterized by a gradual decline in the efficiency and accuracy of biological processes, leading to deterioration of physiological functions and development of age-associated diseases. Age-dependent accumulation of genomic instability and development of metabolic syndrome are well-recognized components of the aging phenotype, both of which have been extensively studied. Existing findings strongly support the view that the integrity of the cellular genome and metabolic function can be influenced by light at night (LAN) and associated suppression of circadian melatonin production. While LAN is reported to accelerate aging by promoting age-associated carcinogenesis in several animal models, the specific molecular mechanism(s) of its action are not fully understood. Here, we review literature supporting a connection between LAN-induced central circadian disruption of peripheral circadian rhythms and clock function, LINE-1 retrotransposon-associated genomic instability, metabolic deregulation, and aging. We propose that aging is a progressive decline in the stability, continuity, and synchronization of multi-frequency oscillations in biological processes to a temporally disorganized state. By extension, healthy aging is the ability to maintain the most consistent, stable, and entrainable rhythmicity and coordination of these oscillations, at the molecular, cellular, and systemic levels.

## GENOMIC INSTABILITY, ITS SOURCES, AND IMPACT ON AGING

Genomic instability is a hallmark of many human diseases with cancer and progeroid syndromes representing the most common outcomes associated with the loss of genome integrity (Anisimov, 2003; Campisi, 2005; Coppede and Migliore, 2010; Vijg and Suh, 2013). The link between genomic instability, cancer, and aging is not surprising as accumulation of mutations resulting in clinically relevant tumors takes time. Further evidence for the importance of cancer prevention for extended longevity resides in the resistance of long-lived rodents to spontaneous and induced tumorigenesis. These exceptional animals exemplify unique evolutionary adaptations preventing cancer development (Gorbunova et al., 2012; Tian et al., 2013). Humans also possess genes positively associated with longevity (Jazwinski et al., 2010; Kim et al., 2012), and exceptionally long-lived individuals typically do not develop cancer, often despite practicing unhealthy lifestyles. This is probably achieved by assuring the fidelity of DNA damage repair, which normally declines with age (Gorbunova et al., 2007).

Genomic instability arises from either exogenous or endogenous sources. Numerous exogenous carcinogenic agents (IR, UV, heavy metals, cigarette smoke, etc.) are well recognized. Artificial LAN represents a recent and unique addition to the list of genome offenders. Night shift work, which is regarded in epidemiological studies as a surrogate for LAN, involving circadian disruption has been recognized as a probable carcinogen (class 2a) by the World Health Organization (Bonde et al., 2012; Stevens et al., 2013, 2014). While the mechanistic relationship between this environmental factor and genomic instability is not well understood, its negative effect on genome integrity is substantiated by the fact that LAN increases cancer risk in humans and promotes aging and cancer growth in animal models (Davis et al., 2001; Schernhammer et al., 2001; Anisimov et al., 2004; Megdal et al., 2005; Vinogradova et al., 2009; Zhu et al., 2009; Wu et al., 2011).

Among established endogenous sources of DNA damage are reactive oxygen species (ROS), stalled replication forks, replication errors, and mitochondrial dysfunction. Much research has been dedicated to understanding their origin and their contribution to aging (Vijg and Suh, 2013). Another, frequently overlooked, source of endogenous genomic instability are transposable elements. These entities, which are present in most analyzed genomes, can rearrange the genetic material of their hosts in the process of their mobilization (reviewed in Belancio et al., 2010a). While their role in aging and cancer has long been debated, the empirical evidence for their actual involvement in these processes has only recently begun to accumulate (Gasior et al., 2006; Belancio et al., 2010b; Evrony et al., 2012; Lee et al., 2012; Solyom et al., 2012; De Cecco et al., 2013).

Genomic instability manifests itself in different ways. Single base-pair substitutions or deletions are the smallest genetic changes that can completely abolish gene function when they occur at positions critical for gene expression or activity. Another type of mutation is large genomic rearrangements such as deletions, insertions, inversions, and translocations, often referred to as chromosomal instability. They commonly result from the misrepair of DNA double-strand breaks (DSBs), which can be caused by stalled replication forks or external and endogenous DNA damaging agents. While all types of mutations are known to contribute to tumorigenesis, the rate and the spectrum of their accumulation with age demonstrates significant variation and tissue-specificity (Vijg and Dolle, 2002). Large genomic deletions, rather than point mutations, are believed to contribute to the aging phenotype as they are more likely to perturb regulation of gene expression, leading to accumulation of dysfunctional mosaic cells in aging tissues (Vijg and Dolle, 2002; Hsieh et al., 2013).

The spectrum and rate of accumulation of mutations can be greatly affected by genotype and environmental exposures with many, seemingly independent, cellular processes, and external factors influencing genome stability. Among the relevant genes are those involved in DNA repair, circadian regulation, and metabolism (Fu et al., 2002; Grimaldi et al., 2010; Kang et al., 2011; Gotoh et al., 2014). The pathways they specify can be disrupted or altered by various environmental cues such as LAN and diet. Not surprisingly, mutations abrogating these pathways lead to increased genomic instability and age-associated diseases. The majority of proteins involved in DNA repair, metabolism, and circadian pathways are highly conserved among evolutionarily distant organisms, further underscoring the fundamental importance of maintaining DNA integrity. Thus, the genetic, metabolic, and environmental effects on aging can be considered in the context of interconnected entities of the same system, synchronized with its environment, rather than individual, autonomous pathways. Here, we discuss emerging connections between genomic instability, transposable elements, circadian regulation, and metabolism.

## RETROELEMENTS AND AGING

Retroelements are mobile genetic entities that are a universal feature of many evolutionarily diverse organisms (reviewed in Belancio et al., 2008). Only 25% of the genome of the naked mole rat, a long-lived rodent, is occupied by transposon-derived repeats compared to 40% in human, 37% in mouse, and 35% in rat (Keane et al., 2014). Retroelements belong to two evolutionarily related groups of LTR (long terminal repeat) and non-LTR retrotransposons. In mammals, they are represented by endogenous retroviruses and Long and Short Interspersed Elements (LINEs and SINEs) and SVA elements, respectively (reviewed in Belancio et al., 2008). Non-LTR retroelements amplify through a “copy-and-paste” mechanism, which has allowed them to amass to over 500,000 copies per genome (Lander et al., 2001; Bibillo and Eickbush, 2002). LINEs, SINEs, and SVA are the only retrotransposons currently active in the human genome (Lander et al., 2001).

L1 elements can contribute to genomic instability through the retrotransposition of themselves and their parasites Alu and SVA (Moran et al., 1996; Dewannieux et al., 2003; Hancks et al., 2011; Raiz et al., 2012), as well as by induction of DSBs (Gasior et al., 2006; Belancio et al., 2010b; Kines et al., 2014). Both types of damage rely on the function of the endonuclease domain (EN) of the L1 ORF2 protein (Feng et al., 1996). EN is responsible for breaking genomic DNA to initiate *de novo* integration. The L1 ORF2p also possesses a reverse transcriptase (RT) domain, which functionally connects L1 to all RT-using entities (Mathias et al., 1991).

Historically, L1 activity was believed to be restricted to the germ line, early embryogenesis, and transformed somatic cells. The discovery of endogenous L1 mRNA expression in normal human tissues opened the possibility of L1 involvement in aging (Belancio et al., 2010b), and the report of endogenous L1 mobilization within human cortex and caudate neurons (Evrony et al., 2012) provided the first direct evidence of L1 activity in normal cells. Next generation sequencing (NGS) conducted at the single cell level showed a rate of 0.04–0.07 somatic L1 inserts per neuron. With an estimated 100 billion neurons per human brain and 50 trillion cells per human body, this finding suggests that there are about 4 billion neurons containing somatic L1 inserts in an average human brain and millions of *de novo* L1 integration events in every normal individual.

While some understanding of the rate of L1 retrotransposition *in vivo* is emerging, the amount of damage associated with L1-induced DSBs remains unknown. Some evidence exists that DSBs associated with L1 activity are 10–100 times more frequent than *de novo* L1 integrations (Gasior et al., 2006), suggesting that L1 may be responsible for 0.4–7 DSBs per cell. DSBs are one of the most harmful lesions in mammalian cells, because they are typically mutagenic when misrepaired by the NHEJ repair pathway (Gorbunova and Seluanov, 2005). DSB-induced mutations and unrepaired DSBs are known to accumulate with age (Vijg and Dolle, 2002; Sedelnikova et al., 2004). DSBs can be toxic to mammalian cells when unrepaired. Consistent with this notion, transient L1 overexpression in primary normal human cells and stem cells leads to apoptosis or senescence (Belancio et al., 2010b). This could potentially be one of the reasons for detection of low L1 retrotransposition *in vivo*, as normal cells supporting high L1 activity may be efficiently eliminated. L1-induced senescence of adult stem cells could contribute to their depletion with age. All of the above suggest that L1 may be responsible for the generation of mutations reported to accumulate with age as well as for promoting cellular senescence which is reported to increase with age (Jeyapalan et al., 2007).

The estimated L1 insertion frequency reflects retrotransposition in a cellular environment with all mechanisms in place to suppress these elements. There is a continually growing list of mammalian genes that negatively regulate different steps of the L1 replication cycle (reviewed in Belancio et al., 2008). Even though most of these have not yet been validated *in vivo*, their increasing number and the diversity of the pathways reported to control L1 activity underscore the necessity of their efficient suppression. It has been hypothesized that genomes deficient in cellular functions critical for L1 downregulation are burdened with higher rates of L1-induced genomic instability. For example, most human cancers support higher L1 expression than the normal tissues from which they have originated (Bratthauer and Fanning, 1992; Bratthauer et al., 1994). Recent NGS studies of L1 retrotransposition in human cancers provide experimental support for higher *de novo* L1 mobilization in human tumors relative to normal somatic tissues (Lee et al., 2012; Solyom et al., 2012; Tubio et al., 2014). As most human cancers harbor defects in many DNA repair or DDR pathways, these findings suggest that the rate of *de novo* L1 retrotransposition in normal tissues may increase with acquired elimination or age-associated decline of negative regulators of the L1 replication cycle. The significant redundancy of pathways suppressing L1 activity suggests that increased L1 mobilization may occur with sequential inactivation of multiple pathways or through circadian disruption of a “master regulator” controlling multiple cellular processes.

**FIGURE 1:**
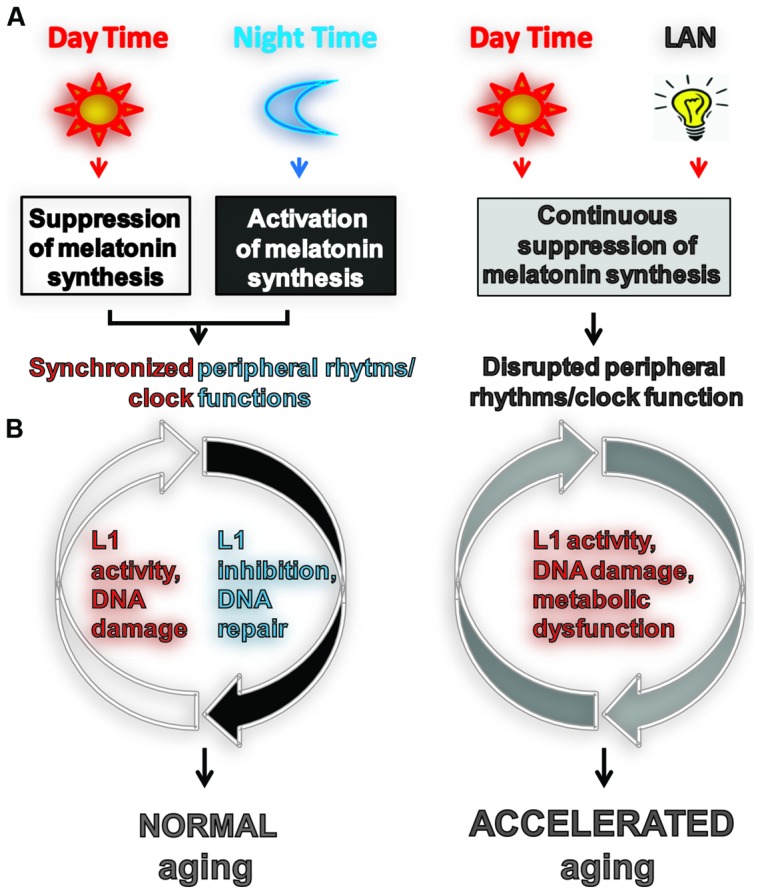
**Light exposure at night accelerates aging by impeding or enhancing processes associated with aging. (A)** Usually aging involves normal light exposure that is characterized by alternating intervals of light and dark over a 24-h period, which result in circadian production of nocturnal melatonin. This leads to the synchronization of peripheral clock (PC) function controlling many biochemical processes in cells including L1 expression and activity (Deharo et al., 2014) and the DNA damage response (DDR). **(B)** Exposure to light at night (LAN) is reported to accelerate aging. LAN blocks nocturnal melatonin production which prevents synchronization of PCs, leading to the disruption of timely function of many biochemical processes in cells including L1 expression and activity, DDR, and metabolism.

There are several tangible connections between L1 activity and the host circadian system (**Figure 1**). One of the recently discovered factors suppressing L1 is melatonin signaling (Deharo et al., 2014). Activation of melatonin receptor 1 (MT_1_) suppresses L1 expression in an *in vivo* cancer model and dramatically decreases L1 retrotransposition in cultured cells. This connects the activity of an endogenous DNA damaging entity with a component of the host circadian system and with the periodicity of environmental light exposure. In addition to its direct effect on L1 through downregulation of L1 ORF1 protein, melatonin signaling is also involved in the synchronization of uniquely timed biochemical functions in peripheral tissues and their associated cellular clock mechanisms which include the DDR. This suggests that circadian disruption may also indirectly increase L1 activity via deregulation of pathways important for the suppression of these elements. The best example is the reported circadian regulation of the nucleotide excision repair (NER) pathway activity in mice (Kang et al., 2009), which is also a suppressor of L1 retrotransposition in cultured cells (Gasior et al., 2008).

## CIRCADIAN CONNECTION BETWEEN AGING, METABOLISM, AND GENOME STABILITY

The functions performed by individual cells are coordinated with the activity of their neighboring and distant cells by the circadian system (Dibner et al., 2010). The circadian system typically contains three major components: a central clock (CC), an entrainment pathway(s), and CC-responsive peripheral tissues and their associated peripheral clocks (PCs). The CC is located in the hypothalamic suprachiasmatic nucleus (SCN) of the brain and it is often referred to as the “master clock” because of its autonomous nature. The autoregulatory activity of the CC provides temporal organization of rhythmic function of PCs and thus many molecular processes in somatic tissues. While self-sufficient, the CC is entrained (synchronized) by external stimuli which is essential for adaptation of various functions within organisms in anticipation of daily changes in their environment.

The most potent external stimulus influencing the activity of the CC is environmental light/dark cycle. Daily periodicity of the light/dark cycle synchronizes the CC-driven oscillation of melatonin production in the pineal gland. Melatonin is a neurohormone that is produced during the dark phase of the 24 h light/dark cycle. Melatonin is an ancient and evolutionarily conserved molecule that is found in animals, plants, and microbes. Its main role in mammals is to inform the CC and all peripheral cells (including their endogenous clockworks) of the onset of nighttime (darkness) and to initiate actions associated with the nighttime of the daily cycle (Pfeffer et al., 2012). Like light during the daytime, melatonin during the nighttime helps to reset the CC in mammals. Melatonin functions through its G-protein coupled receptors MT_1_ and MT_2_ both of which are expressed in the CNS and peripheral tissues (Masana and Dubocovich, 2001; Poirel et al., 2003). Interestingly, the nocturnal mode of melatonin production is the same in both nocturnal and diurnal animals even though they exhibit inverse times of their sleep/wake activity (Dauchy et al., 2010). Despite these behavioral differences, the disruption of the circadian melatonin signal in both leads to the same negative effects on their health (Vinogradova et al., 2009), supporting an important, sleep-independent role of melatonin on health.

Melatonin production can be easily disrupted by LAN which commonly occurs in shift workers (Lewy et al., 1980; Blask et al., 2011). Melatonin synthesis also declines with age (Reiter et al., 2002; Hill et al., 2010; Zhdanova et al., 2011; Baba et al., 2012). LAN-induced melatonin suppression is associated with metabolic dysfunction, obesity, and an increased risk of several malignancies (Anisimov et al., 2004; Blask et al., 2005, 2011, 2014; Lai et al., 2009; Vinogradova et al., 2009; Wu et al., 2011). While the effect of LAN on human aging is not known, the LAN-induced shortening of life span in rodent models supports the existence of a biological connection between CC and PC functions, genomic instability, metabolism, and aging (Reiter et al., 2002; Wood et al., 2009; **Figure 1**). Recent findings have demonstrated a direct relationship between the biological clock, aging, and sphingolipid metabolism, arguing for a conserved circadian-based mechanism of aging from fungi to humans (Case et al., 2014).

Metabolic cycles are tightly coupled with both CC and PCs allowing both diurnal and nocturnal mammalian species to coordinate nutrient use and storage with light/dark entrained sleep/wake cycles in the overall regulation of organismal bioenergetics (Bass, 2012). Metabolically active tissues (e.g., liver, adipose tissue, skeletal muscle) are highly responsive to circadian oscillations in circulating glucose, fatty acids, triglycerides, and metabolic hormones (Bellet and Sassone-Corsi, 2010). Circadian clocks control several critical metabolic pathways and, conversely, metabolic processes exert important feedback effects on the molecular clock machinery. A significant player in the bidirectional interactions between circadian signaling and metabolic activities is thought to be nicotinamide adenine dinucleotide (NAD^+^) which functions as an electron shuttle in oxidoreductase reactions. Additionally, NAD^+^ is a critical cofactor for sirtuins, most notably SIRT1, which regulate metabolism in response to caloric restriction and as modulators of oxidative damage and DNA repair processes that appear to be critical for lifespan. The circadian regulation of NAD^+^-dependent sirtuin activity may have implications for healthy aging and oxidative metabolism that is particularly relevant to the association of circadian period length and longevity (Bass, 2012; Masri and Sassone-Corsi, 2014). Further support for the presence and conservation of this mechanism comes from the discovery that human Sirt1 and six modulate the function of circadian clock genes (Asher et al., 2008; Jung-Hynes et al., 2010; Chang and Guarente, 2013; Masri et al., 2014) and the expression of Sirt1 and some clock genes in normal tissues is enhanced by melatonin (Chang et al., 2009; Yu et al., 2014).

**FIGURE 2:**
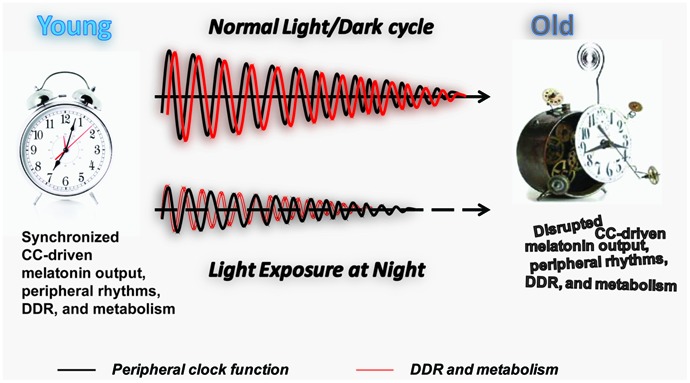
**Longitudinal effect of LAN on PC function, DDR, and metabolism.** Schematic representation of the effect of normal light exposure versus LAN on the age-associated deterioration of PCs. The maintenance of the normal light/dark cycle promotes circadian melatonin output and synchronization of the PC (black line) with DDR and metabolic function (red line). An age-associated decline in melatonin production and melatonin receptor expression (Hill et al., 2010) leads to the gradual decline in the amplitude of the peripheral rhythms and potentially their synchronization with DDR and metabolic function. We hypothesize that LAN accelerates aging by promoting age-associated decline in the amplitude of the peripheral rhythms and their synchronization with DDR and metabolic function at early age. Individual genomes may provide molecular machinery to resist adverse effects of LAN, explaining the variation in lifespan observed in the human population.

Melatonin is a powerful antioxidant that suppresses ROS (Reiter et al., 2010) and through its receptor downregulates L1 retrotransposons (Deharo et al., 2014), both of which may elicit DNA damage in the absence of melatonin. Suppression of melatonin signaling is also known to impair cell signaling pathways important for DNA repair, apoptosis, and cellular differentiation (Xiang et al., 2008; Hill et al., 2009; Blask et al., 2011). There is growing support for a connection between systemic DDR, which could be caused by L1 activity, and attenuation of p53 function and metabolic changes, consistent with the age-dependent and LAN-induced decline in DDR efficiency and development of metabolic syndrome caused by decline in circadian clock activity (Erol, 2010; Feng et al., 2012). Furthermore, caloric restriction delays age-associated decline in melatonin production in rhesus monkeys (Roth et al., 2001) and resets circadian rhythms in mice (Froy et al., 2008). In contrast, high fat diet disrupts the normal circadian cycle (Eckel-Mahan et al., 2013). Interestingly, DNA damage itself can reset the circadian clock (Gamsby et al., 2009) suggesting the possibility that controlled periodicity of intrinsic DNA damage in tissues with a synchronized clock may facilitate its maintenance (**Figure 2**). On the other hand, deregulation of intrinsic DNA damage response caused by LAN may further promote desynchronization of PCs. Thus, melatonin signaling is positively associated with molecular functions that activate cellular pathways involved in the maintenance of genome stability and metabolism that are central to healthy aging. Collectively, current data suggest that LAN-induced disruption of the CC and the associated disruption of the PC activate L1-associated DNA damage and metabolic changes in normal tissues, which may contribute to the LAN-induced acceleration of aging manifested in age-associated changes and diseases.

## CONCLUDING REMARKS

Aging has largely been discussed as a complex, but for the most part, linear progression from the beginning to the end of life. The multitude of differences between convenient experimental and simplistic approaches and the actual complexity of life as we age in a continuously changing environment (Chang et al., 2009; Yu et al., 2014) forces us to refine this view. We propose that during aging there is a progressive loss of synchronized oscillation of biological processes along the axis of life accompanied by a continuous decline in their amplitude (**Figure 2**). By extension, healthy aging is the ability to maintain the most consistent light/dark entrainable rhythmicity and coordination at the molecular, cellular, and systemic levels throughout the lifespan, originating in the genetically programmed resistance to environmental cues and stress capable of disrupting this balanced progression. This definition has the potential to explain the puzzling coexistence of an unhealthy lifestyle with an exceptionally long survival.

## Conflict of Interest Statement

The authors declare that the research was conducted in the absence of any commercial or financial relationships that could be construed as a potential conflict of interest.
